# A Case of Extirpation of the Rectum by the Kraske Method

**Published:** 1894-07

**Authors:** X. O. Werder

**Affiliations:** Pittsburg


### A CASE OF EXTIRPATION OF THE RECTUM BY THE KRASKE METHOD.

By DR. X. O. WERDER, Pittsburg.

I wish to present to you a brief report of an extirpation of the rectum by the sacral method, or Kraske operation. The patient, John C., 43 years old, was perfectly healthy until about eight months ago, when he was seized with a very profuse hemorrhage from his bowels. A month or so later he had considerable straining at stool, with bloody discharges, returning at short intervals. These attacks were treated as dysentery. Between these paroxysms of tenesmus, accompanied by mucous and bloody discharges, he had normal evacuations.

When I saw him in consultation, January 11th of this year, I found on the interior wall of the rectum a mass, beginning about one and one-half inches above the external sphincter, of oval shape, and involving about half of the lumen of the rectum; its upper margin could hardly be reached by the examining finger. The examination was not particularly painful and did not cause much bleeding. There could be no doubt as to the nature of the neoplasm, and extirpation of the rectum by Kraske's method was advised.

The operation was performed January 16, 1894, in Mercy Hospital. The patient was placed upon his left side, in Sims's position. An incision from the point near the anus was carried to the middle of the sacrum, the soft parts detached from the left side of the bone. The sacro-sciatic ligament divided, and then the coccyx completely enucleated, and a section of the fifth, fourth and third sacral vertebræ removed with bone forceps. The whole rectum was now fully exposed and the organ separated from its attachments. As these were exceedingly firm, at least over the anterior wall, in which the neoplasm was situated, the enucleation was tedious and very difficult, and accompanied by considerable bleeding. After all adhesions around the rectum had been broken up, a ligature was placed around it above the cancerous growth, and the whole organ removed, including the external sphincter. The artificial anus was attached to the upper angle of the wound, immediately under the remaining portion of the sacrum, by sutures. The wound over the sacrum and coccyx united by sutures, and the pelvis cavity firmly packed with iodoform gauze, the ends allowed to protrude at the lower angle of the wound where the anus had been removed. The patient stood the operation well and rallied nicely from the shock. His recovery was uninterrupted. He never suffered much pain, and the highest temperature was 101⅘°. His bowels were allowed to move on the seventh day. He was able to leave his bed at the end of the third week, and left the hospital five weeks after the operation. He is feeling perfectly well. The only inconvenience he experiences is the fact of not having control of his bowels. He is wearing an apparatus similar to a truss worn for an umbilical hernia, which closes the artificial anus temporarily. Unfortunately I was ignorant at the time of the operation of Gursuny's device of twisting the end of the bowels, which is said to give a sphincter-action to the artificial anus, thereby obviating the unpleasant result of such operation, *i. e.*, loss of control over the action of the bowels.—*Pittsburg Medical Review.*

